# Elevation-dependent shifts in soil phosphorus forms and phosphorus-solubilizing microbial diversity suggest enhanced bioavailable phosphorus cycling with rising temperatures

**DOI:** 10.1128/spectrum.01300-24

**Published:** 2025-07-09

**Authors:** Chuifan Zhou, Xingjian Dun, Qian Tang, Yuntian Long, Jing Du, Mingzhuo Bao

**Affiliations:** 1 Co-Innovation Center for Sustainable Forestry in Southern China of Jiangsu Province, Key Laboratory of Soil and Water Conservation and Ecological Restoration of Jiangsu Province, Nanjing Forestry University 659822, Nanjing, China; 2 Shandong Academy of Forestry 604267, Jinan, China; 3 Fujian Agriculture and Forestry University 12449, Fuzhou, China; University of Mississippi, University, Mississippi, USA

**Keywords:** vegetation type, organic phosphorus mineralizers, elevation gradient, phosphorus forms, global change

## Abstract

Phosphorus is a crucial nutrient for plant growth, primarily found as insoluble phosphates in soil. The impact of climate change on the phosphorus cycle, particularly through geochemical cycling in forest soils, is underexplored. To address this gap, we investigated soil phosphorus distribution across five elevation gradients in different forest types using the modified Hedley fractionation method and high-throughput sequencing to assess phosphorus-solubilizing microorganisms (PSMs). Our study included evergreen broadleaf forest, coniferous and broadleaf mixed forest, coniferous forest, subalpine dwarf forest, and alpine meadow. We observed that with increasing elevation, organic phosphorus forms such as NaOH-Po and HCl-Po significantly increased, while H_2_O-Po and NaHCO_3_-Po varied, peaking in specific forest types. The proportion of labile phosphorus decreased with elevation, whereas stable phosphorus increased. Soil PSM diversity was highest in lower elevations and declined with altitude, with significant shifts in dominant PSM groups across vegetation types. PSMs analysis revealed correlations between specific phosphorus forms and microbial taxa, indicating that elevation and vegetation type influence PSM diversity and phosphorus form distribution. The differences in soil conditions between low and high elevations present distinct characteristics: rapid mineral weathering and organic matter decomposition occur at lower elevations, while higher elevations exhibit higher total phosphorus content but a lower proportion of labile phosphorus. These distinctions open avenues for future research on adaptive strategies for phosphorus management in agriculture and forestry across different altitudes. These findings suggest that rising temperatures may enhance PSM diversity, potentially increasing the proportion of bioavailable phosphorus and accelerating its cycle in forest ecosystems.

**IMPORTANCE:**

This study provides critical insights into how climate change influences soil phosphorus cycling in forest ecosystems. By examining the distribution of phosphorus forms and the diversity of phosphorus-solubilizing microorganisms (PSMs) across different elevation gradients, we uncover how varying environmental conditions affect phosphorus availability. Our findings reveal that higher elevations, despite having increased total phosphorus content, exhibit reduced bioavailable phosphorus. This suggests that climate change, particularly rising temperatures, could enhance PSM diversity at lower elevations, potentially increasing phosphorus bioavailability and accelerating its cycle. These insights are crucial for developing adaptive phosphorus management strategies in agriculture and forestry, ensuring sustainable nutrient supply and improving ecosystem resilience in the face of global climate change. Understanding these dynamics helps predict future soil fertility patterns, aiding in the formulation of policies and practices aimed at maintaining healthy forest ecosystems and ensuring long-term ecosystem productivity under changing climatic conditions.

## INTRODUCTION

Phosphorus (P) is a crucial nutrient for plant growth and the maintenance of ecosystem functions, essential for energy transduction, DNA and RNA synthesis, and cell membrane construction. Its importance is particularly pronounced in subtropical regions where the prevalence of iron and aluminum oxides results in phosphorus forms that are largely inaccessible to plants (1, 2). This phenomenon makes phosphorus bioavailability a limiting factor for vegetative growth and ecosystem productivity in these areas.

In this context, phosphate-solubilizing microorganisms (PSMs) play a critical role. Through the secretion of organic acids and other metabolic products, PSMs convert insoluble soil phosphorus into forms that are bioavailable, thereby enhancing the soil’s accessible phosphorus content (3). Also, as a gene encoding alkaline phosphatase (ALP), *phoD* plays a crucial role in the mineralization of organic phosphorus by *phoD*-harboring microbial communities, which is a significant factor in enhancing soil phosphorus (P) availability (4, 5). This process positions PSMs as vital intermediaries between soil phosphorus availability and plant phosphorus needs, emphasizing their role in linking soil geochemical processes with biotic phosphorus demand.

The impact of global climate change, a subject of significant international concern, on ecosystem dynamics has increasingly come under scrutiny (6–8). Notably, changes in climate, especially temperature increases, are recognized to affect soil nutrient cycling processes. The interaction of the soil phosphorus cycle with climate variables has attracted considerable scientific interest, influencing both plant phosphorus uptake and demand and the viability and community composition of PSMs (9). These changes indirectly impact the availability of soil phosphorus and, consequently, plant nutritional status (10, 11).

The Wuyi Mountains, with their unique geographical positioning and rich biodiversity, provide an unparalleled opportunity for research (7, 12–14). The environmental gradient created by varying altitudes offers a distinctive setting to investigate the responses of PSMs to changes in temperature, humidity, and soil physicochemical properties, acting as a natural laboratory for studying these processes. An in-depth examination of the dynamics of soil PSMs across different elevational zones in the Wuyi Mountains and their role in phosphorus form transformation could reveal patterns of soil phosphorus transformation under the influence of climate change. These studies are crucial for understanding the adaptive mechanisms of soil forms and PSMs to elevational gradients, thereby improving our knowledge of phosphorus cycling within mountainous forest ecosystems (13).

Building upon this foundation, our research posits several hypotheses aimed at deepening the understanding of the interplay between PSMs, soil phosphorus dynamics, and climate change effects within the ecological context of the Wuyi Mountains. We hypothesize that the diversity of phosphate-solubilizing microorganisms (PSMs) decreases with increasing elevation due to the cooler temperatures and distinct soil chemical properties found at higher altitudes. This reduction in diversity may lead to a shift in the dominant PSM species and, consequently, a change in phosphorus solubilization efficiency. Additionally, we propose that the distribution of phosphorus forms in the soil is closely linked to the composition and activity of PSM communities, with elevated PSM diversity at lower elevations contributing to a higher proportion of labile phosphorus forms. Conversely, higher elevations may show an increased prevalence of stable phosphorus due to reduced microbial activity. In addition, we anticipate that the effects of climate change, in particular rising temperatures, will amplify the impacts of increased elevation, which may alter nutrient cycling and ecosystem equilibrium. Through this research, we aim to unveil the resilience and adaptability of PSMs and soil phosphorus dynamics to environmental changes, offering fresh insights into ecosystem responses to global climate shifts. This study seeks not only to contribute to the scientific discussion on phosphorus cycling in mountainous regions but also to provide guidance for effective conservation and soil management strategies amidst changing climatic conditions.

## MATERIALS AND METHODS

### Study area overview

The study area is located within the Wuyi Mountain National Park in the northwestern part of Fujian Province, China (27°33′N to 27°54′N, 117°27′E to 117°51′E). This region falls within the humid subtropical monsoon climate zone, with an annual average precipitation of 2,000 mm (13, 14). The geomorphology is predominantly hills and high mountains, with a clear vertical zonation of soil types, including mountainous red soil, yellow-red soil, yellow soil, and alpine meadow soil. Vegetation types vary with elevation, in order (Fig. S1): Evergreen Broad-leaved Forest (EB), Coniferous and Broad-leaved Mixed Forest (CB), Coniferous Forest (CF), Sub-alpine Dwarf Forest (SD), and Alpine Meadow (AM) (7, 12). The specifics of the study area are described in our previous study (7).

### Soil sampling

Within each vegetation zone’s corresponding altitude range (EB: 300 m, CB: 1,000 m, CF: 1,400 m, SD: 1,700 m, AM: 1,960 m), three naturally similar plots were selected for replication, using a handheld GPS device (Explorist 610, Magellan, USA) to determine the precise location and elevation. In each vegetation zone, three 20 m × 20 m plots were established as replicates, with 30–50 m between each plot. Within each plot, one sampling point was selected at regular intervals in an “S” shape, for a total of six sampling points, and soil was collected 20 cm below the surface on 19 January 2019 (winter) after removal of the dead litter layer. After collection, soil from the six points was mixed equally to form one sample per plot. A total of 15 composite samples were collected from five different locations, with visible roots, stones, and other debris removed. Part of each composite sample was air-dried at room temperature and sieved through a nylon mesh for the determination of phosphorus components and phosphatase activity. Another portion of the soil sample was sieved through a 2 mm mesh and stored at −80°C for PSMs analysis (3).

### Phosphorus form fractionation method

Total phosphorus (TP) in the soil samples was determined via digestion with 10 mL of H_2_SO_4_ added to 0.25 g of the sample. The concentration of phosphorus in all extracts was measured using the molybdenum blue method and a UV-visible spectrophotometer (T6, Puxi, China) at 700 nm, with blank extractions performed for available phosphorus, total phosphorus, and values subtracted from the sample extracts. Soil phosphorus was fractionated using the modified Hedley method, dividing it into five groups: H_2_O-P (H_2_O-Po and H_2_O-Pi), NaHCO_3_-P (NaHCO_3_-Po and NaHCO_3_-Pi), NaOH-P (NaOH-Po and NaOH-Pi), HCl-P (HCl-Po and HCl–Pi), and residual-P (15, 16). Total P (TP) was measured after acidifying the supernatants with ammonium persulfate at 121°C for 1 hour. TP and Pi were measured directly from the extracts, while Po was calculated as the difference between TP and Pi.

### Soil phosphatase and PSMs sequencing methods

Soil phosphatase activity was determined using the method by Eivazi and Tabatabai (17). The hydrolysis of phenyl disodium phosphate into phenol and hydrogen disodium phosphate was catalyzed by acid phosphatase (ACP) as well as alkaline phosphatase (ALP) in acidic and alkaline environments, respectively. The phosphatase activity was calculated by measuring the amount of phenol produced. PSMs sequencing was carried out by Beijing Allwegene Technology Co., Ltd. (Beijing, China), extracting DNA from soil samples, then amplifying the *phoD* gene using specific primers (F733: 5′-TGGGAYGATCAYGARGT-3′; R1083: 5′-CTGSGCSAKSACRTTCCA-3′) in a thermal cycler. The temperature and cycling conditions were as follows: preheating at 94°C for 5 min, followed by 37 cycles at 94°C for 30 s, 50°C for 30 s, and 72°C for 60 s, and a final incubation at 72°C for 7 min. The amplification products were detected by electrophoresis on a 1% agarose gel and sequenced using the Illumina MiSeq platform (3, 18). The sequencing data has been submitted to the NCBI BioProject database under the accession number PRJNA1171756. Detailed data processing methods are provided in Supplementary Material S1.

### Data processing and statistical analysis

Statistical analysis was performed using SPSS v.19.0, conducting one-way ANOVA on data for soil PSMs, phosphatase activity, and phosphorus components. Means displaying significant differences were compared using the Least Significant Difference (LSD) post-hoc test (*P* < 0.05). Principal Component Analysis (PCA) calculated microbial community evolutionary distances between samples using the Bray-Curtis coefficient, with differences among multiple samples described by an Unweighted Pair Group Method with Arithmetic Mean (UPGMA) clustering tree. We employed the “linkET” package to draw Mantel-r correlation heatmaps (19). We used the “randomForest,” “rfpermute,” and “plspm” packages to analyze Random Forest Models and Partial Least Squares Path Modeling (PLS-PM) in R v4.1.3 (R Core Team, 2018) software to explore the relationships among soil altitude, dominant bacterial phyla, phosphorus forms, enzyme activity, and diversity (20, 21).

## RESULTS

### Variation in total phosphorus content, phosphorus fraction content, and their ratios across different vegetation types

**Fig 1:**
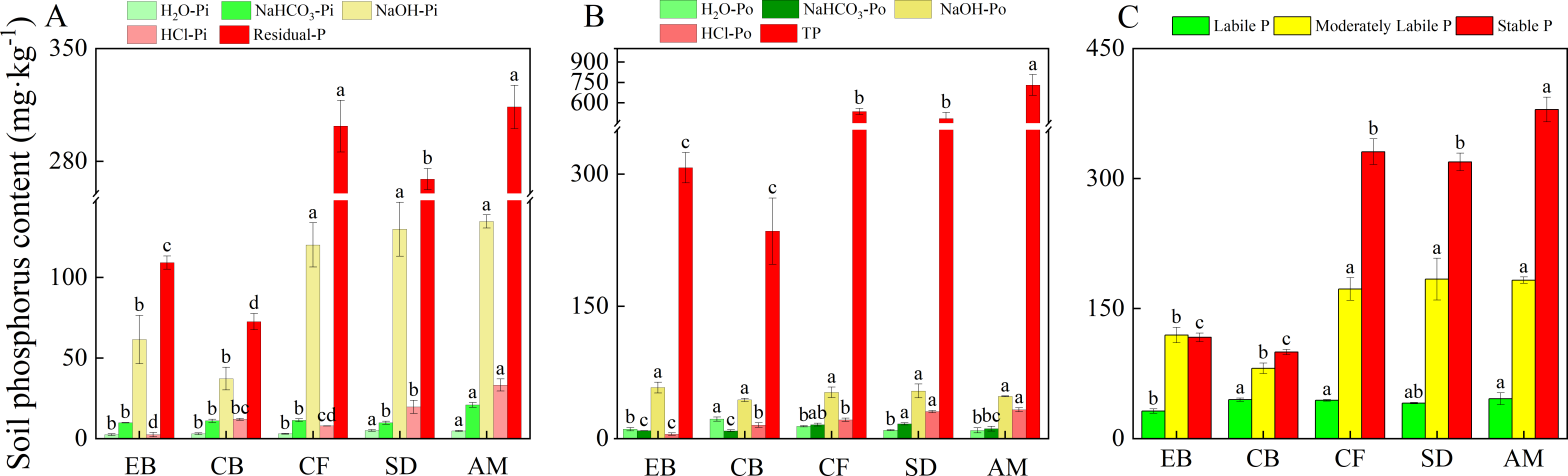
The content of inorganic phosphorus (**A**), organic phosphorus, and total phosphorus (**B**),labile, moderately labile and stable phosphorus (**C**) across different vegetation types. Note: Different lowercase letters at the top represent significant differences in the content of the same phosphorus fraction between sites (*P* < 0.05), the same below.

The content of inorganic phosphorus in soil increased with the elevation gradient, becoming significantly different starting from CF. Despite a decrease in NaHCO_3_-Pi content within CB, it was not significantly different from EB (Fig. 1A). Significant differences in soil total phosphorus (TP) content were observed between different vegetation types (*P* < 0.05), with an increasing trend alongside elevation. Among these, the TP content in the Alpine Meadow (AM) was higher than in other vegetation types, with Coniferous Forest (CF) and Sub-alpine Dwarf Forest (SD) significantly higher than Evergreen Broad-leaved Forest (EB) and Coniferous and Broad-leaved Mixed Forest (CB) although no significant changes were observed between the former two (Fig. 1B). Significant differences were detected across all organic soil P fractions among vegetation types, except for NaOH-Po content variation. H_2_O-Po content was highest in CB, while NaHCO_3_-Po content was significantly highest in CF and SD. The content of HCl-Po gradually increased with elevation, significantly increasing in SD and AM (Fig. 1B).

**Fig 2:**
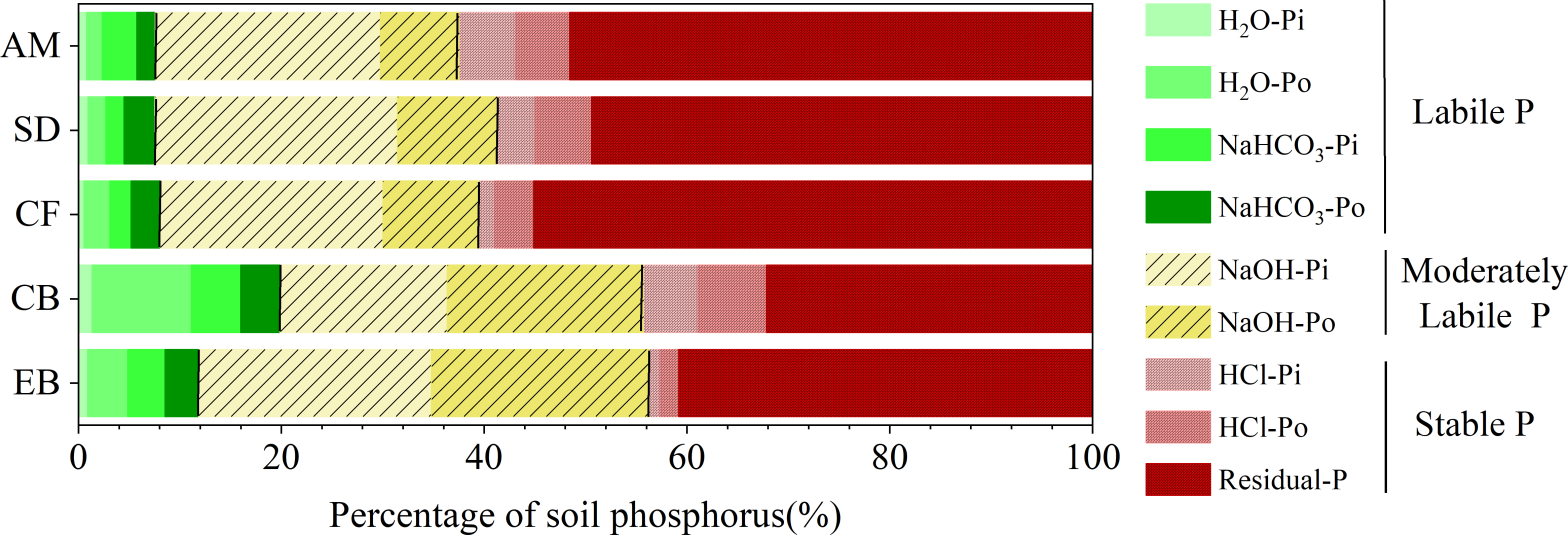
The proportion of phosphorus components in soils of different vegetation types.

The proportion of labile and moderately labile phosphorus decreased significantly with elevation from 56% to 38%, while the proportion of stabilized phosphorus increased significantly from 44% to 62% (Fig. 2).

### Impact of vegetation type on soil enzyme activity

**Fig 3:**
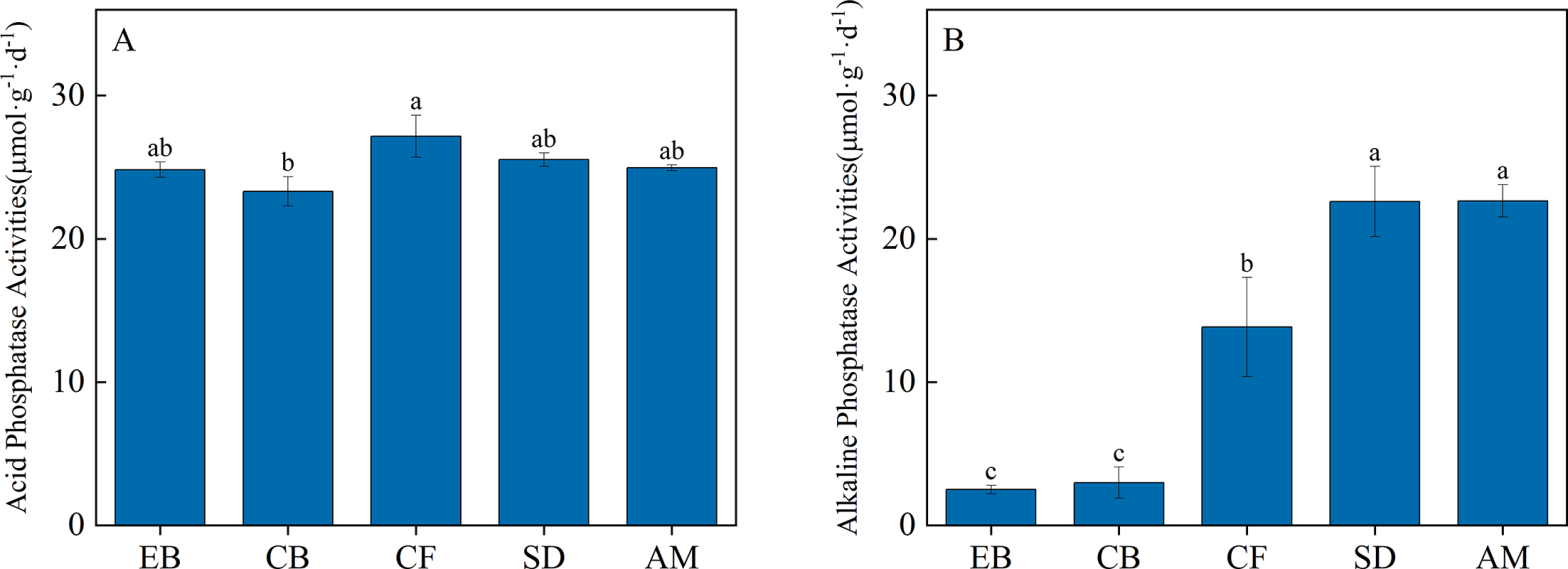
Soil acid phosphatase (**A**) and alkaline phosphatase activities (**B**) across different elevational vegetation types.

Across different vegetation types, acid phosphatase activity was higher than alkaline phosphatase activity. Except for CF, where acid phosphatase activity was significantly higher than in CB, no significant differences were observed in acid phosphatase activity among the other vegetation types (Fig. 3A). Soil alkaline phosphatase activity increased with increasing elevation gradient, significantly increasing from 2.49 µmol·g^−1^·d^−1^ at the lowest elevation to 22.65 µmol·g^−1^·d^−1^ at the highest elevation (Fig. 3B).

### Diversity of soil PSMs across different vegetation types

**Fig 4:**
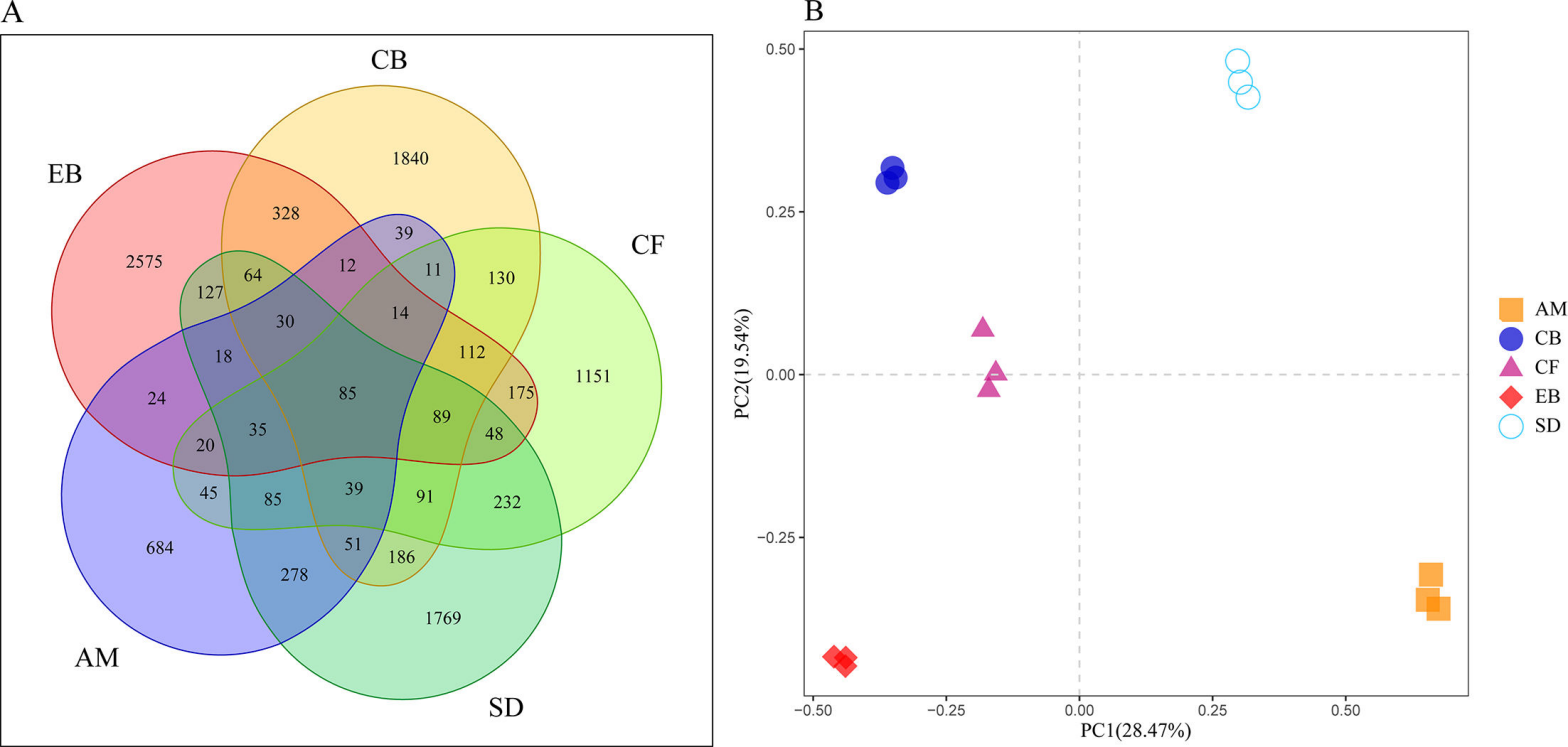
Soil PSMs Venn diagram (**A**) and PCA analysis (**B**) across different elevational vegetation types.

The Venn diagram illustrates the diversity of PSM across different vegetation types (Fig. 4A). The number of OTUs in EB, CB, CF, SD, and AM were 3,756, 3,121, 2,362, 3,227, and 1,470, respectively, with EB having the highest number of OTUs (Fig. 4A). Additionally, EB had the highest number of unique OTUs (2,575), indicating a more specific type of PSM within this vegetation type. PCA analysis also showed differences in community composition across different vegetation types (Fig. 4B), with the first principal component (PC1) contributing 28.85% and the second principal component (PC2) contributing 20.65%. The distances analyzed correspond to the differences between vegetation types, suggesting that differences in forest types lead to variations in soil PSM composition (Fig. 4B). PCA reflects that the resolving distance between CB and CF is relatively close, indicating similar soil PSM structures in these two types.

**Fig 5:**
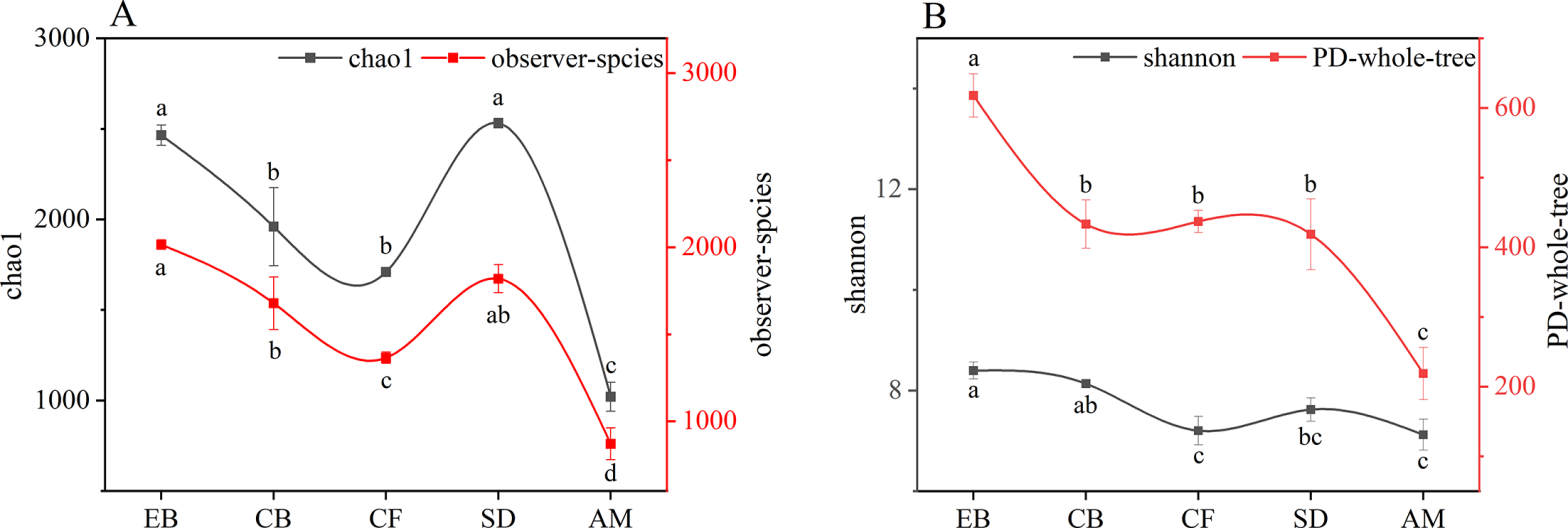
Diversity analysis of soil PSMs across different elevational vegetation types. Note: (A) Black line and black *Y*-axis indicate chao1 index; red line and red *Y*-axis indicate observer-species index; (B) Black line and black *Y*-axis indicate Shannon index; red line and red *Y*-axis indicate PD-whole-tree index.

As elevation increases, excluding SD, the overall trend of diversity indices decreases. Indices such as PD-whole-tree, Shannon index, Chao1, observed species number, and Chao1 values were highest in EB and lowest in AM, indicating that PSMs diversity indices and species numbers are highest at lower elevations and lowest at higher elevations (Fig. 5).

### Composition of dominant soil PSMs across vegetation types

**Fig 6:**
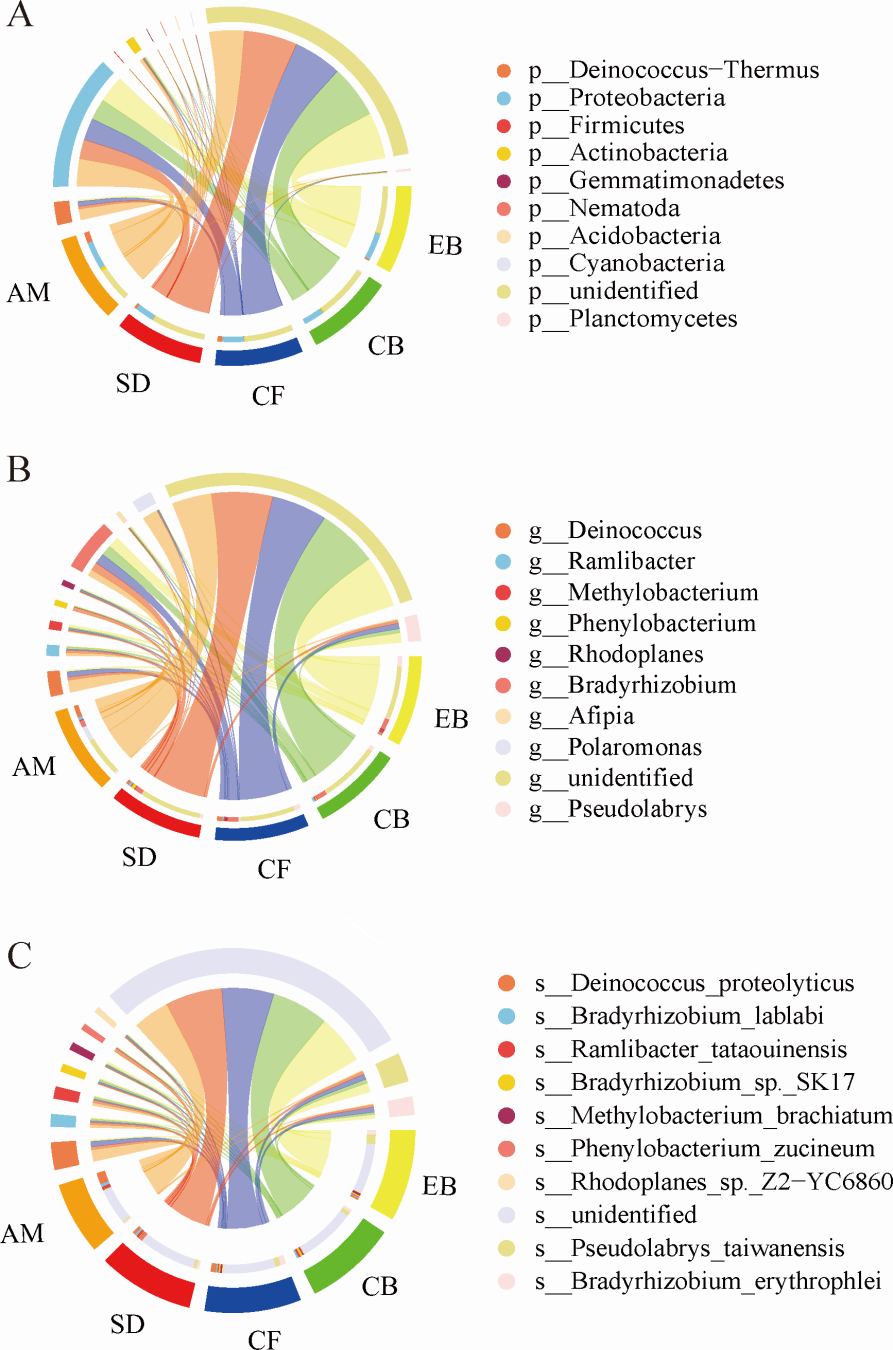
Composition of soil PSMs across different elevational vegetation types at the phylum (**A**), genus (**B**), and species levels (**C**). Note: Five sections are distinguished below each chord diagram, each representing a different environment, with different colored sectors in each section showing the relative abundance of microorganisms in that environment; the legend lists the microorganisms corresponding to each color at the top of the chord diagram, and the thickness of the arcs indicates the magnitude of the abundance of the same microorganism in different environments.

At the phylum level, the dominant PSM were *Proteobacteria*, *Deinococcus-Thermus*, *Actinobacteria*, and *Planctomycetes. Proteobacteria* were consistently dominant across all vegetation types without significant variation. The relative abundance of *Actinobacteria* and *Firmicutes* in AM was significantly higher than in other vegetation types. The increase of *Deinococcus-Thermus* was most significant in CF and AM (Fig. 6).

At the genus level, *Bradyrhizobium* and *Pseudolabrys* had the largest proportions across all vegetation types. The relative abundance of *Polaromonas* in AM and *Phenylobacterium* in SD was significantly higher than in other vegetation types. However, *Pseudolabrys*, *Methylobacterium*, and *Ramlibacter* showed an opposite pattern. The relative abundance of *Deinococcus* increased with elevation (Fig. 6). At the species level, dominant species such as *Pseudolabrys taiwanensis* and *Bradyrhizobium erythrophlei* in EB showed a significant decreasing trend with elevation, while *Deinococcus proteolyticus*, *Polaromonas* sp. *SP1*, and *Bradyrhizobium lablabi* showed a significant increasing trend and became dominant species in AM soil.

### Relationship between PSMs, phosphatases, and phosphorus forms

**Fig 7:**
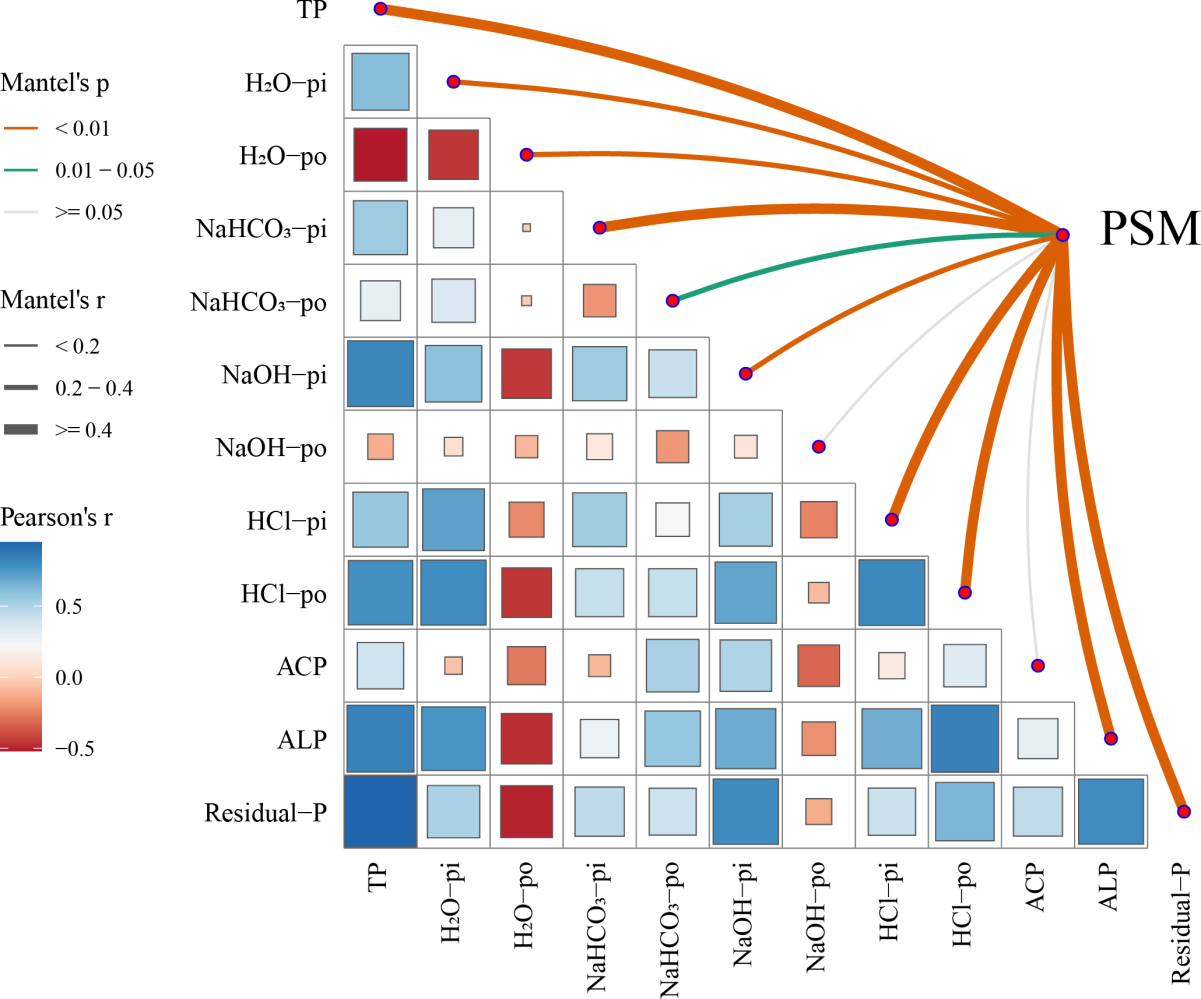
Mantel *r* analysis of superimposed heat maps. Note: Rectangular frames denote correlations between different environmental factors established using Pearson’s method, where blue represents positive correlations, and red indicates negative correlations. The deeper the color, the more significant the correlation. The larger the box, the stronger the correlation. The *P*-value (*P*.value) and Mantel_*r* value collectively reflect the impact of environmental factors on the phosphorus-solubilizing bacterial community. Smaller *P*-values and larger Mantel_*r* values suggest a greater influence of environmental factors on phosphorus-solubilizing bacteria. Different ranges of *P*-values are represented by lines of different colors in the figure (dashed lines indicate negative correlations, and solid lines indicate positive correlations), whereas the Mantel_*r* values are denoted by the thickness of the lines.

Mantel_r analysis on soil phosphorus forms and PSMs revealed that PSMs have a highly significant positive correlation with TP, H_2_O-Pi, H_2_O-Po, NaHCO_3_-Pi, NaHCO_3_-Po, NaOH-Pi, HCl-Pi, HCl-Po, alkaline phosphatase (ALP), and Residual P (*P* < 0.05), with the greatest impact (*r* value) on HCl-Pi, HCl-Po, ALP, and Residual P (Fig. 7).

**Fig 8:**
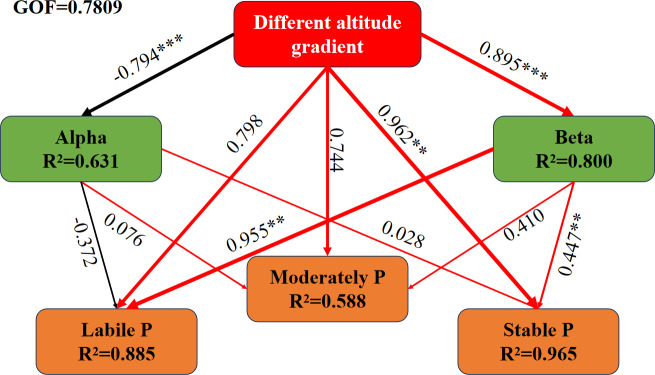
Partial least squares path modeling (PLS-PM) showing the relationship between altitude gradient and phosphorus. Note: *, **, and *** indicate significant correlations at the 0.05, 0.01, and 0.001 levels, respectively. Line thickness in the figure represents the absolute value of the direct effect of path coefficients, with red lines indicating positive effects and black lines indicating negative effects.

PLS-PM showed the relationship between soil PSMs alpha diversity and beta diversity, soil phosphorus forms, and their impact on active phosphorus, moderately labile phosphorus, and stable phosphorus under different elevational gradients (Fig. 8). The Goodness of Fit (GOF) was 0.78. It was observed that different elevational gradients have a significant negative correlation with PSMs alpha diversity , while showing significantly positive correlations with the beta diversity of PSMs and stable phosphorus. The beta diversity of PSMs was significantly positively correlated with both stable phosphorus and active phosphorus. However, the alpha diversity and beta diversity of PSMs showed no significant correlation with moderately active phosphorus.

**Fig 9:**
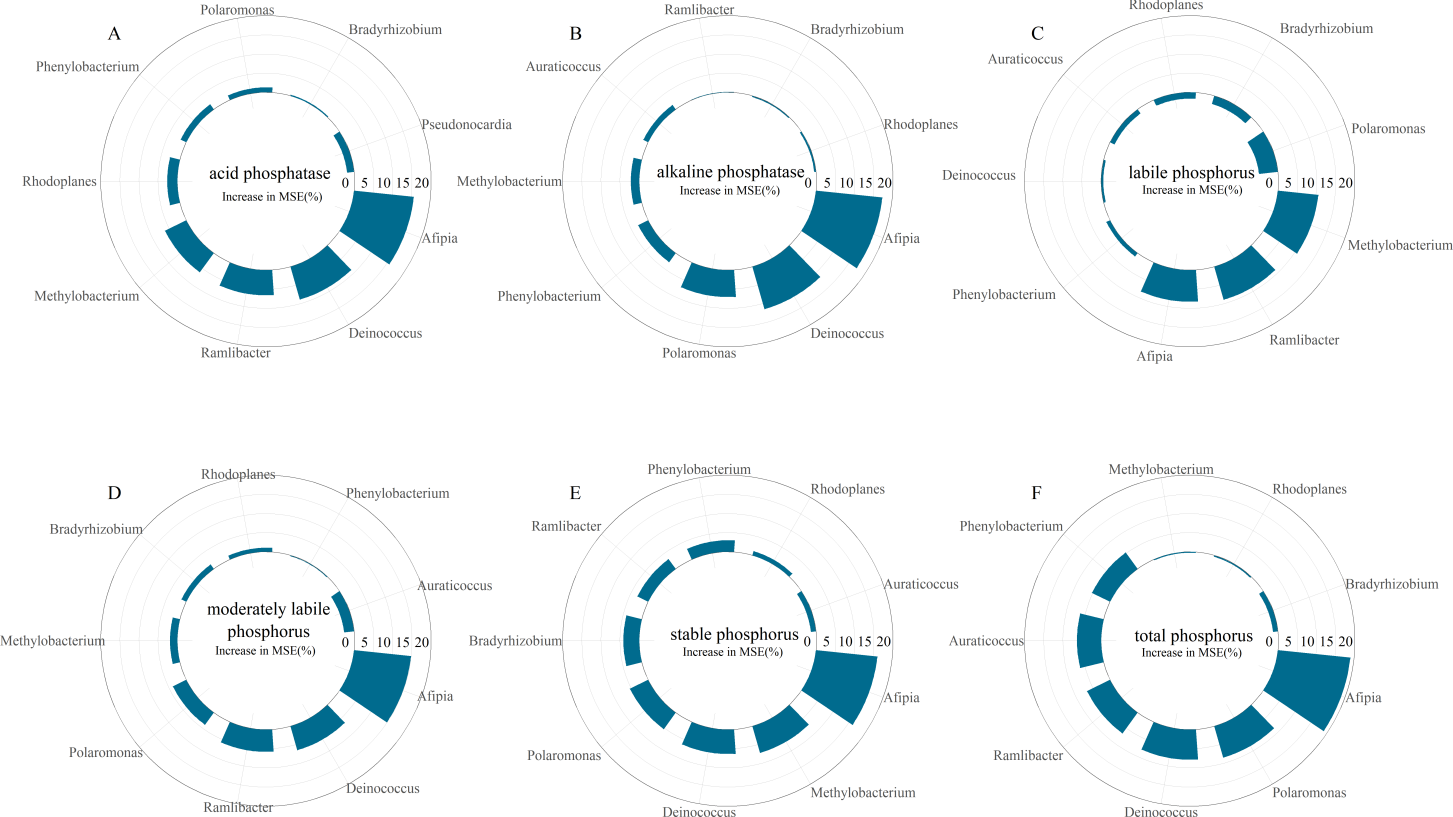
Evaluation of the impact of PSM on phosphorus transformation and enzyme activity at the genus level using a random forest model. Acid phosphatase (**A**), alkaline phosphatase (**B**), active phosphorus (**C**), moderately active phosphorus (**D**), stable phosphorus (**E**), and total phosphorus (**F**).

The Random Forest model (Fig. 9) showed the importance of soil dominant PSMs on acid phosphatase (A), alkaline phosphatase (B), labile phosphorus (C), moderately labile phosphorus (D), and stable phosphorus (E), total phosphorus (F) (increase in MSE%). *Deinococcus*, *Rhodoplanes*, *Bradyrhizobium*, *Phenylobacterium*, *Ramlibacter*, *Methylobacterium*, *Auraticoccus*, *Afipia*, and *Polaromonas* were identified as the top nine dominant PSM genera, with *Afipia* having the greatest impact on various soil phosphorus forms and enzyme activities (Fig. 9). *Deinococcus* and *Ramlibacter* also played important roles in total phosphorus (Fig. 9F), enzyme activity (Fig. 9A and B), and stable phosphorus (Fig. 9E), while *Methylobacterium* had a greater impact on labile phosphorus than on moderately labile and stable phosphorus (Fig. 9C through E). This indicates that the dominant PSM groups play a crucial role in the phosphorus transformation process across different elevations in the Wuyi Mountains.

## DISCUSSION

### Phosphorus forms and transformation dynamics across altitudes

Our findings indicate a general increase in total soil phosphorus (TP) content with elevation, a phenomenon particularly pronounced in alpine meadows (AM) relative to evergreen broadleaf forests (EB). This observation corroborates previous research (22, 23), suggesting that increased soil TP content at higher elevations may be governed by a complex interplay of climatic conditions, soil properties, and vegetation types. Factors including climate change, modifications in soil characteristics, and variations in organic phosphorus contributions across different vegetation types likely serve as major drivers collectively contributing to the observed upward trend in soil TP levels.

In the soil matrix, intrinsic transformations occur among different phosphorus forms, adhering to the hierarchical trend: stable phosphorus > moderately labile phosphorus > labile phosphorus (Fig. 2) (24). Stable phosphorus primarily encompasses Residual-P and HCl-P. Residual-P refers to a highly stable form of phosphorus in soil, which remains unextractable by chemical reagents due to its tight binding to soil particles or organic matter, or its incorporation within the mineral lattice, rendering it less accessible for direct plant uptake. HCl-P mainly consists of phosphorus occluded by iron hydroxide films (O-P). Moderately labile phosphorus, primarily represented by NaOH-P, is chemically adsorbed onto the surfaces of iron (Fe) and aluminum (Al) oxides (15).

With increasing elevation, the content of stable, moderately labile, and labile phosphorus generally shows an upward trend; however, the proportion of labile phosphorus decreases. This pattern aligns with the intensification of soil weathering due to temperature variations, leading to the mineralization of primary mineral and organic phosphorus, thereby decreasing the total and bioavailable phosphorus content with rising temperatures or decreasing elevation. For instance, soils in evergreen broadleaf forests (EB) at lower elevations are subjected to higher temperatures and lower soil pH levels, which facilitate mineral weathering and organic matter decomposition (7, 25). This accelerates the mineralization of organic phosphorus, releasing more bioavailable phosphorus (26). Conversely, at higher elevations, increased litterfall and organic matter content reflect the altitudinal trend in soil organic phosphorus content, indicating that litter quantity and organic matter are significant factors influencing soil phosphorus content (7).

### Role of phosphate-solubilizing microorganisms in phosphorus cycling

The activity of microorganisms is pivotal in augmenting the release of insoluble phosphorus in soils, with phosphate-solubilizing microorganisms (PSMs) serving as a vital component in this biochemical process. PSMs possess the capability to transform insoluble phosphorus compounds—such as organic phosphorus and phosphates bound with iron—into soluble forms, thus enhancing the bioavailability of phosphorus for plant uptake. This biochemical transformation is crucial for sustaining plant nutrition and maintaining ecosystem productivity.

Our research revealed that soils from evergreen broadleaf forests (EB) and conifer-broadleaf mixed forests (CB) exhibited higher percentages of labile phosphorus, accompanied by the lowest contents of stable phosphorus (Fig. 2). Different α-diversity indices reflect the diversity of PSMs in different ways. The Shannon index reflects both species evenness and species abundance. The Chao1 index, on the other hand, reflects species richness and changes in rare species (27, 28). observed_species is the number of OTUs actually observed with increasing sequencing depth. pd_whole_tree is a diversity index that takes into account species abundance as well as evolutionary distance (29). The four different diversity indices were higher in the soils of EB and CB, indicating that PSM in the soils of EB and CB showed the highest diversity and species richness (Fig. 5). In addition, we showed through PLS-PM that different altitudinal gradients significantly affect the diversity of PSM composition and community structure. Liable phosphorus is the main factor affecting PSM beta diversity, while soil phosphorus activation can recruit more different types of PSM and improve the efficiency of phosphorus conversion and utilization. This observation underscores the significant role that PSMs play in the conversion of stable phosphorus forms into labile phosphorus, facilitating the dynamic phosphorus cycling within these ecosystems.

The findings are in concordance with the research by Zhang (3), which demonstrated a positive correlation between increased PSM diversity and elevated levels of active soil phosphorus. This relationship is marked by a concomitant decrease in the proportion of stable phosphorus, highlighting the transformative impact of PSMs on phosphorus bioavailability. The presence of a diverse and abundant PSM community suggests that these microorganisms not only enhance the solubilization of phosphorus but also influence the overall soil phosphorus dynamics by potentially altering the soil’s chemical equilibria through the secretion of organic acids and other metabolites.

Furthermore, the intricate interactions between PSMs and soil phosphorus availability illustrate the microorganisms’ adaptability and their pivotal role in linking geochemical processes with biotic nutrient demands. This underscores the necessity of incorporating microbial dynamics into broader ecological models that aim to predict and manage nutrient cycles under varying environmental conditions and anthropogenic pressures. Understanding these microbial processes is essential for developing strategies to optimize phosphorus use efficiency in both natural and agricultural systems, especially in the context of global climate change which may alter the environmental parameters that govern microbial activity and diversity (30).

### Implications of altitudinal variation on soil PSM diversity and phosphorus availability

Moreover, with increasing altitude, there is a general decline in the diversity and abundance of soil phosphate-solubilizing microorganisms (PSMs) (Fig. 5), a trend that aligns with findings from previous studies (10, 31–33), which report decreased microbial diversity at higher elevations. It is estimated that over 80% of PSMs secrete organic acids such as malic, citric, lactic, propionic, gluconic, succinic, oxalic, and tartaric acids (34, 35). These organic acids not only reduce soil pH, thereby facilitating the dissolution of insoluble phosphates, but also chelate with ions such as iron, aluminum, and calcium, thus releasing phosphate ions into the soil matrix.

In addition, the activity of phosphatases, which is intimately linked to the abundance and vigor of the soil microbial community, allows PSMs to hydrolyze organic phosphorus substrates into inorganic phosphorus, thereby enhancing soil phosphorus availability (36, 37). Moreover, PSMs form symbiotic associations with plant roots, facilitating the conversion of phosphorus into forms that are readily accessible to plants and thereby improving plant phosphorus uptake. The presence of PSMs can modify soil microbial community structure and soil conditions, thereby stimulating the activity of other soil microorganisms and promoting both the degradation of organic phosphorus and the broader process of phosphorus cycling (38). Collectively, these mechanisms underscore the critical role of PSMs in enhancing soil phosphorus availability and promoting plant phosphorus absorption and utilization.

Further analysis has identified the principal phyla of PSMs within the soils of the Wuyi Mountains as *Proteobacteria, Deinococcus-Thermus*, *Actinobacteria*, and *Planctomycetes* (Fig. 6A). These findings are consistent with subtropical research conducted by Hu et al. (39). *Proteobacteria* and *Actinobacteria*, characterized as copiotrophic bacteria, are classified into ecological strategies; *Proteobacteria* are r-strategists that rely on organic matter for metabolic energy. They flourish in environments with high soil carbon content or lower pH levels, thus promoting the survival and proliferation of r-strategist bacteria (40, 41). Conversely, *Actinobacteria*, which function as K-strategists, are integral to soil organic matter formation, aiding in organic matter decomposition, and have phosphorus-solubilizing capabilities although their growth and reproduction can be restricted in environments with excessively high organic matter content (39, 40).

Certain PSMs, especially those belonging to the *Deinococcus-Thermus* group—such as *Deinococcus* species, *Deinococcus proteolyticus*, *Polaromonas sp*. SP1, and *Bradyrhizobium lablabi*—demonstrate a notable increase with elevation. The *Deinococcus-Thermus* group is renowned for its exceptional resistance to harsh environmental conditions, including radiation, oxidation, desiccation, and low temperatures, thereby playing a significant role in augmenting phosphorus availability at high altitudes (1, 31, 39). *Deinococcus* species, typically chemolithoautotrophic, can thrive on nutrient-poor media as well as standard media, with strains exhibiting a broad range of temperature tolerances (42, 43). Consequently, the increased diversity and richness of PSM species are significant contributors to the higher proportion of active phosphorus in soils at lower elevations.

### Conclusion

Our investigation into the variations of phosphorus forms across different elevations reveals a consistent trend: as elevation increases, the content of all phosphorus forms escalates, with a hierarchy observed from stable phosphorus (primarily Residual-P and NaOH-P), through moderately labile phosphorus, to labile phosphorus. This gradient effect underscores the intricate balance and transformation dynamics within the phosphorus cycle, highlighting the decrease in the proportion of labile phosphorus at higher elevations. The discernible, significant correlations between various phosphorus forms at different elevations not only underscore the complex interplay and intrinsic transformations among them but also point toward the nuanced impact of elevation on phosphorus bioavailability in soil. Our study also sheds light on the pivotal role of phosphate-solubilizing microorganisms (PSMs) in this context, with a noted decline in PSM diversity and abundance at higher elevations. The shift in dominant PSM phyla—*Proteobacteria*, *Deinococcus-Thermus*, *Actinobacteria*, and *Planctomycetes*—with increasing altitude, provides critical insights into the adaptability and resilience of microbial communities to altitude-induced environmental stresses. This observation suggests a link between microbial diversity and the soil phosphorus cycle, influencing the availability of phosphorus for plant uptake across varying ecological zones. Innovatively, our research highlights the altitude-driven dichotomy in phosphorus content and microbial diversity, contributing to the broader understanding of soil phosphorus dynamics and microbial ecology. The differences in soil conditions between low and high elevations present distinct characteristics: rapid mineral weathering and organic matter decomposition occur at lower elevations, while higher elevations exhibit higher total phosphorus content but a lower proportion of labile phosphorus. These distinctions open avenues for future research on adaptive strategies for phosphorus management in agriculture and forestry across different altitudes. The extrapolation of results across broader geographical scales and diverse ecological settings requires caution due to the complex interplay of climatic, biotic, and abiotic factors influencing phosphorus cycling and microbial diversity. Additionally, the role of specific PSMs in mediating phosphorus solubilization and their interaction with plant root systems at varying elevations warrants further investigation to elucidate the mechanisms underlying phosphorus availability and uptake. Future research should focus on expanding the scope of microbial and phosphorus analyses to a wider range of elevations and ecosystems, incorporating longitudinal studies to assess the impact of climate change on soil phosphorus dynamics and microbial ecology. Furthermore, exploring the potential of bioengineering PSMs to enhance phosphorus solubilization and availability in phosphorus-limited soils presents an exciting frontier for sustainable agricultural practices and ecological conservation.

## SUPPLEMENTAL MATERIAL

Supplemental figures and tables spectrum.01300-24-s0001.docx Figure S1 and S2, and Table S1.

ASM does not own the copyrights to Supplemental Material that may be linked to, or accessed through, an article. The authors have granted ASM a non-exclusive, world-wide license to publish the Supplemental Material files. Please contact the corresponding author directly for reuse.
