# Supplementary material for: Elevation-dependent shifts in soil phosphorus forms and phosphorus-solubilizing microbial diversity suggest enhanced bioavailable phosphorus cycling with rising temperatures
Source: Microbiol Spectr. 2025 Jul 9;13(8):e01300-24. doi: 10.1128/spectrum.01300-24 (PMC12323630; doi:10.1128/spectrum.01300-24)
Supplement: Supplemental figures and tables — Figure S1 and S2, and Table S1. [file spectrum.01300-24-s0001.docx]

***Fig. S1 Schematic diagram of the sample plots***


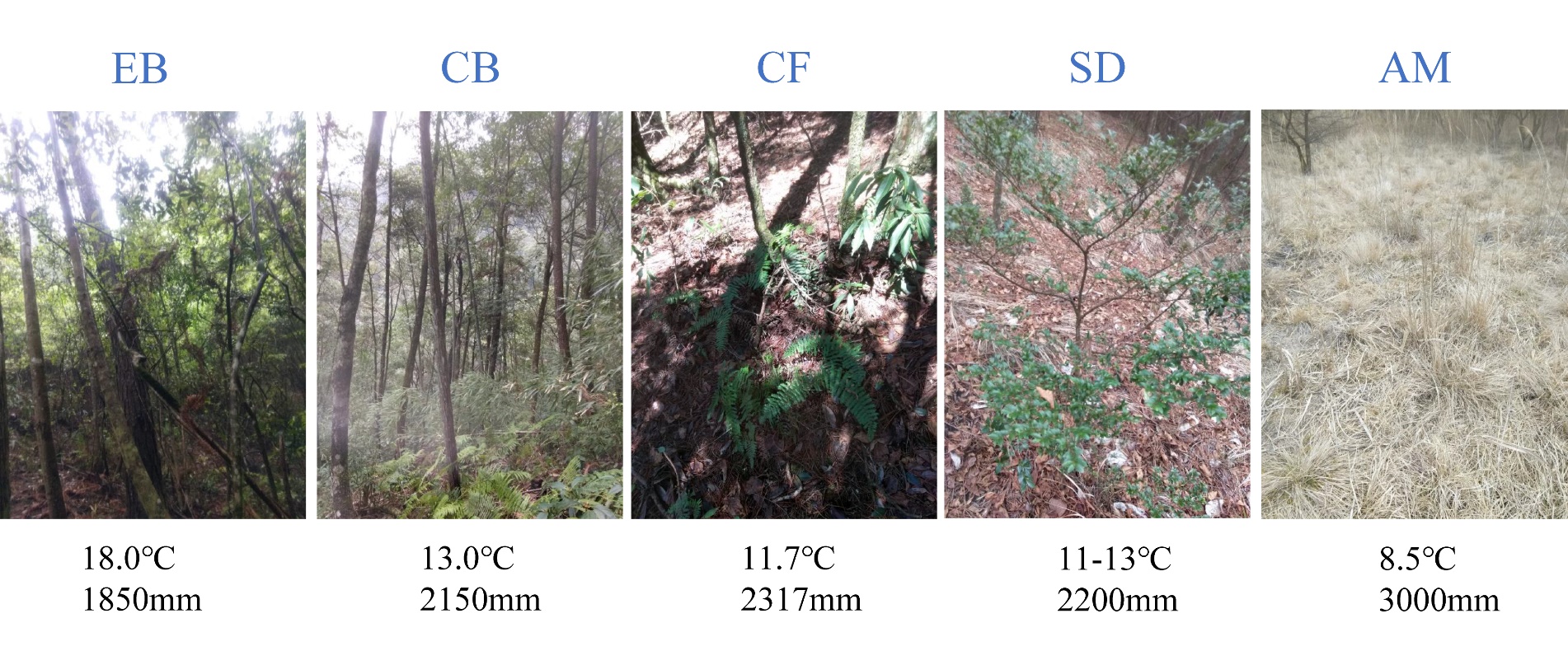


Note: Evergreen Broad-leaved Forest (EB), Coniferous and Broad-leaved Mixed Forest (CB), Coniferous Forest (CF), Sub-alpine Dwarf Forest (SD), and Alpine Meadow (AM); In addition the figure contains temperatures at different sites as well as precipitation amounts.

***S1 PSMs Sequencing Data Processing Methods***

Miseq sequencing generates paired - end (PE) sequence data. The Fastq data is subjected to quality control using Trimmomatic (v0.36) (1) and Pear (v0.9.6) (2). For Trimmomatic, a sliding window strategy is adopted. The window size is set to 50bp, the average quality value is 20, and the minimum retained sequence length is 120. Pear is used to remove sequences containing N. Flash (v1.20) (3) and Pear are employed to merge the two - end sequences according to the overlap relationship of PE. The minimum overlap is set to 10bp, and the mismatch rate is 0.1, resulting in Fasta sequences. The uchime method is used to align and remove chimeras from the Fasta sequences based on known databases. For unknown databases, the denovo method is used for self - alignment and removal of chimeras. Sequences that do not meet the requirements are also removed (4). The downcomer data (Raw PE), after removing barcode and primer and splicing to get raw_tags, raw_tags after further removing chimeras, short sequences to get quality sequence clean_tags. The clean_tags are then clustered into OTUs using the uparse method (5). To obtain the taxonomic information corresponding to each OTU, the RDP Classifier algorithm is employed to align and analyze the representative sequences of OTUs (6). The community's taxonomic information is annotated at various levels (kingdom, phylum, class, order, family, genus, species).

***S2 Four Different α-diversity Indices***

The Shannon index is a widely used diversity index that takes into account both species richness and evenness (7).

$$H^{'}=-\sum_{i=1}^{s} p_{i}ln \left( p_{i} \right)$$

where *s* is the total number of species, and *p_i_* is the relative abundance of the *i*-th species (i.e., the proportion of individuals of that species to the total number of individuals). The higher the Shannon index value, the greater the species diversity.

Chao1: Also known as the species richness index, it is used to estimate the number of OTUs in a community (8).

$$\text{Schao1}=\text{Sobs}+\frac{n1\left( n1-1 \right)}{2\left( n2+1 \right)}$$

where Schao1 is the estimated number of OTUs, Sobs is the observed number of OTUs, *n*1 is the number of OTUs with only one sequence, and *n*2 is the number of OTUs with only two sequences.

Observed species: The observed number of species is a straightforward diversity index that simply counts the actual number of different species observed in a specific area.

PD_whole_tree: Phylogenetic diversity is a diversity index that takes into account both species abundance and evolutionary distance (9, 10). It is a diversity index calculated based on a phylogenetic tree. It uses the distances of the phylogenetic tree constructed from the representative sequences of OTUs in each sample. The sum of the branch lengths of all representative sequences in a particular sample is calculated to obtain the value. The higher the value, the greater the community diversity.

***References***

1. Bolger AM, Lohse M, Usadel B. 2014. Trimmomatic: a flexible trimmer for Illumina sequence data. Bioinformatics 30: 2114-2120
2. Zhang J, Kobert K, Flouri T, Stamatakis A. 2014. PEAR: a fast and accurate Illumina Paired-End reAd mergeR. Bioinformatics 30: 614-620
3. Magoč T, Salzberg SL. 2011. FLASH: fast length adjustment of short reads to improve genome assemblies. Bioinformatics 27: 2957-2963
4. Edgar RC, Haas BJ, Clemente JC, Quince C, Knight R. 2011. UCHIME improves sensitivity and speed of chimera detection. Bioinformatics 27: 2194-2200
5. Edgar RC. 2013. UPARSE: highly accurate OTU sequences from microbial amplicon reads. Nature Methods 10: 996-998
6. Wang Q, Garrity GM, Tiedje JM, Cole JR. 2007. Naïve Bayesian Classifier for Rapid Assignment of rRNA Sequences into the New Bacterial Taxonomy. Applied and Environmental Microbiology 73: 5261-5267
7. Shannon CE. 1948. A mathematical theory of communication. The Bell System Technical Journal 27: 379-423
8. Chao A. 1984. Non-parametric estimation of the classes in a population. Scandinavian Journal of Statistics 11: 265-270
9. Faith DP. 1992. Conservation evaluation and phylogenetic diversity. Biological Conservation 61: 1-10
10. Caporaso JG, Kuczynski J, Stombaugh J, Bittinger K, Bushman FD, Costello EK, Fierer N, Peña AG, Goodrich JK, Gordon JI, Huttley GA, Kelley ST, Knights D, Koenig JE, Ley RE, Lozupone CA, McDonald D, Muegge BD, Pirrung M, Reeder J, Sevinsky JR, Turnbaugh PJ, Walters WA, Widmann J, Yatsunenko T, Zaneveld J, Knight R. 2010. QIIME allows analysis of high-throughput community sequencing data. Nature Methods 7: 335-336

**Table S1** Content of various phosphorus fractions in soils with different vegetation cover

|  | H_2_O-Pi | H_2_O-Po | NaHCO_3_-Pi | NaHCO_3_-Po | NaOH-Pi | NaOH-Po | HCl-Pi | HCl-Po | Residual-P | TP |
| --- | --- | --- | --- | --- | --- | --- | --- | --- | --- | --- |
| EB | 2.37 ± 0.55b | 10.55 ± 1.88b | 9.82 ± 0.17b | 8.86 ± 0.14b | 61.44 ± 14.97b | 57.76 ± 6.19a | 2.37 ± 1.35d | 5.03 ± 1.29c | 109.29 ± 4.10c | 307.10 ± 17.27c |
| CB | 3.01 ± 0.55b | 22.04 ± 2.43a | 10.99 ± 1.05b | 8.76 ± 1.10b | 37.21 ± 7.04b | 43.80 ± 1.83a | 11.93 ± 0.65bc | 15.24 ± 2.62b | 72.60 ± 5.03d | 235.18 ± 37.57c |
| CF | 2.92 ± 0.25b | 13.97 ± 0.76b | 11.36 ± 0.87b | 15.74 ± 1.60a | 120.26 ± 13.77a | 52.27 ± 5.98a | 7.86 ± 0.04cd | 21.25 ± 1.85b | 301.80 ± 16.03a | 535.79 ± 22.61b |
| SD | 4.99 ± 0.51a | 9.54 ± 0.63b | 9.79 ± 0.91b | 16.63 ± 1.28a | 130.06 ± 16.75a | 53.87 ± 7.87a | 19.60 ± 4.09b | 30.53 ± 1.37a | 268.90 ± 6.58b | 483.61 ± 45.28b |
| AM | 4.71 ± 0.32a | 9.27 ± 2.89b | 20.86 ± 1.46a | 11.17 ± 2.81b | 134.82 ± 4.07a | 47.83 ± 0.46a | 33.24 ± 3.75a | 32.64 ± 2.34a | 313.69 ± 13.47a | 730.32 ± 76.97a |

Note: Evergreen Broad-leaved Forest (EB), Coniferous and Broad-leaved Mixed Forest (CB), Coniferous Forest (CF), Sub-alpine Dwarf Forest (SD), and Alpine Meadow (AM); Values in the table are presented through a mean ± standard error format, with different lowercase letters representing significant differences in the same phosphorus fraction under different vegetation covers (*p* < 0.05).
